# Influence of perfluorohexyloctane (Evotears®) on higher order aberrations

**DOI:** 10.1007/s10792-023-02905-w

**Published:** 2023-10-21

**Authors:** Amr Saad, Andreas Frings

**Affiliations:** 1 Department of Ophthalmology, University Hospital Duesseldorf, Heinrich-Heine University, Moorenstraße 5, 40225 Duesseldorf, Germany; 2 Augenheilkunde und Augenlaserzentrum PD Dr. med. Frings Nuremberg, Jena, Germany

**Keywords:** Dry eye, Tear film, Artificial tears, Perfluorohexyloctane, Evotears, Higher order aberrations, Refractive surgery

## Abstract

**Purpose:**

To prospectively assess the effect of regular application of perfluorohexyloctane (F6H8; Evotears®) on the tear film lipid layer, higher order aberrations (HOA) and the repeatability of measurements in healthy eyes.

**Methods:**

This prospective clinical study included 104 eyes treated with F6H8 four times daily for four weeks (group A) and 101 eyes that served as controls (group B). Measurements were performed with the WASCA aberrometer (Carl Zeiss Meditec GmbH, Jena, Germany). Main outcome measurement in addition to subjective refraction were the root mean square values of HOA measured before and after the intervention.

**Results:**

Regular use of F6H8 over a period of four weeks significantly increases HOA in healthy eyes (*p* < 0.05). In addition, the repeatability of measurement increases after the application of F6H8.

**Conclusion:**

F6H8 may be a suitable treatment option to improve the accuracy of refractive assessment, although it increases HOA. Further studies are needed to confirm the effect on HOA and the repeatability of measurement.

## Introduction

Dry eye disease, a multifactorial ocular condition characterized by inadequate tear production presents a complex challenge in ophthalmology [1]. Refractive surgery is known to be a risk factor for dry eye, especially after LASIK procedures, where up to 75% of patients may experience dry eye symptoms [2, 3]. Preoperative evaluation and subsequent treatment of dry eye symptoms prior to refractive surgery is generally recommended with artificial tears (AT) [4]. With many treatment options available today, a differential evaluation of specific AT supplements on the precorneal tear film would provide more evidence for ophthalmologists.

Higher order aberrations (HOA) occur in the presence of disorders of the tear film, which are typical for patients with dry eye disease, especially early after refractive surgery [5]. Previous studies have shown that a uniform reduction of the tear film seems to have little influence on the image quality [6]. However, if local irregularities occur in the precorneal tear film, this can negatively affect optical image quality in terms of HOA [6]. A recently published systematic review confirms the strong association between dry eyes and increased HOA [7]. The instability of the tear film and its premature break-up are usually considered to cause local fluctuations [6]. The HOA associated with dry eyes negatively affects the Quality of Live (QoL) [8, 9]. Nevertheless, it is difficult to compare studies in this context, as the type of eye drops used, the time between the last application, the number of applications and the measuring devices are inconsistent. The influence of the lipid layer on HOAs has not yet been thoroughly studied while more lipid-containing eye drops become readily available [10].

The purpose of this prospective, controlled clinical study was to evaluate the effect of a non-aqueous liquid, perfluorohexyloctane (F6H8; Evotears®), on HOA after four weeks of application in otherwise healthy subjects. We aimed also to assess the repeatability of HoA measurements with and without F6H8 application.

## Materials and methods

This prospective interventional randomized study included healthy individuals without a history of dry eye disease. All participants gave written informed consent for pseudonymized data analysis and anonymous publication during the recruitment process. The study and consent procedure were approved by the ethics committee of the University of Duesseldorf, Germany (no. 6057R), and adhered to the tenets of the Declaration of Helsinki. It was also registered in the “German Clinical Trials Register” (trial number: DRKS00030660).

The study group consisted of 104 healthy individuals (68 females, 36 men, mean age 34 ± 2.8 years) and another 101 individuals as control (55 females, 46 men, mean age 36 ± 4.2 years). Study participants were recruited the same private practice. Exclusion criteria were the application of other eyedrops during the study period, the presence of another previously diagnosed chronic ocular surface disease (e.g., perennial allergic conjunctivitis), previous ocular surgery, or contact lenses wear.

Study participants (group A) were asked to start application of a completely non-aqueous liquid containing only perfluorohexyloctane (F6H8), a semifluorinated alkane (SFA, Evotears® Ursapharm Arzneimittel GmbH, Saarbrücken, Germany), four times daily for four weeks. The control group (group B) did not receive any treatment. To allocate participants into the groups, block randomization was applied.

All examinations were performed at baseline and at four-week follow-up. Study participants were advised to discontinue use of the study medication 12 h prior to measurements. All measurements were performed between 9 and 12 am. Aberrometry assessment using WASCA (Carl Zeiss Meditec GmbH, Jena, Germany) was performed before any other scans or manifest refraction to minimize acquisition time and the possible influence of fatigue on measurement repeatability. Three consecutive scans were acquired under mesopic lighting conditions for the right and then the left eye, by a single optometrist according to a standardized operating procedure, including standardized oral instructions for each patient. We instructed patients to keep their forehead and chin in contact with the rests, to avoid head tilt, to keep their focus relaxed—looking through the fixation target rather than at it—and to blink whenever they wanted, but to keep their eyes wide open in between blinks.

Measurements were performed three seconds after the last blink as suggested by Hagyo et al. [11]. We analyzed the RMS values (root mean square) of HOA, which is the standard deviation of all polynomials, representing the mean deviation of all Zernike coefficients from the ideal wavefront in a single eye [12].

We archived anonymized data extracts in an Excel spreadsheet (Microsoft Corp, Seattle) for analysis and filtered outlying values using plausibility limits to screen for data entry errors. In the subset of 100 eyes studied for measurement repeatability, we calculated 95% Limits of Repeatability (95% LoR) from the standard deviation within measures (Sw) derived from a random effects ANOVA applying the formula: 95% LoR = 1.96*SQRT(2)*Sw [13]. We calculated LoR for spherical equivalent (SE) values normalized to a 5 mm pupil for the following variables before and after treatment: Sphere, Cylinder, Coma, Trefoil, Spherical Aberration (SA), and root mean square total higher order aberrations (RMS-HOA). We compared pupil diameters throughout the aberrometry scan acquisition sequence as a surrogate measure of accommodation control and measurement fatigue during scanning. For the first one hundred eyes, we derived Limits of Repeatability (LoR) and bias, or mean difference, values for measured aberrometric and manifest refraction spherical equivalent values [14]. Aberrations were reported in D as there is a linear conversion between μm and D, with no averaging or assumptions.

## Results

Eighty-nine percent of eyes had a mesopic pupil size and aberrometry scan acquisition diameter > 5.0mm. Table 1 summarizes the pre- and post-interventional data. Differences between Coma, Trefoil, SA, and RMS-HOA were statistically significant (*p* < 0.05) signaling an increase of HOA by applying F6H8. The refraction values (SE and Cylinder) did not change significantly. The standard deviation (SD) of the SA and HOA averages was observed to be 1–2.5 times higher than the difference between the pre- and post-treatment values. Data on controls did not yield in statistically significant changes, comparable to data on study group eyes prior to the intervention.

**Table 1:** Refractive data before and after administration of perfluorohexyloctane (F6H8) (*N* = 104 eyes)

Variable	Prior to intervention		After intervention		*p* value
	Mean	SD	Mean	SD	
SE	− 4.271	1.228	− 4.247	1.459	0.781
Cylinder	0.718	0.574	0.780	0.667	0.124
Coma	0.095	0.050	0.140	0.090	0.001
Trefoil	0.094	0.079	0.112	0.088	0.043
SA	0.067	0.066	0.093	0.073	0.001
RMS-HOA	0.272	0.069	0.323	0.076	0.001

Dioptric spherical equivalent values standardized for a 5 mm pupil were applied throughout

*SE* Spherical equivalent, *SA* Spherical aberration, *RMS-HOA* Root mean square higher order aberrations

The mean pre- and post-interventional values for the aberrometric indices and the 95% LoR for the first 100 eyes are tabulated (Table 2). After the intervention, we found a decrease in the 95% LoR for all parameters, indicating that differences between consecutive measurements were more reliable and repeatable after the application of F6H8.

**Table 2:** Measurement repeatability in aberrometry before and after administration of perfluorohexyloctane (F6H8) (*N* = 100 eyes)

	Measurement 1		Measurement 2		Measurement 3		95% rep. limit
	Mean	SD	Mean	SD	Mean	SD	
Variable							
Prior to intervention							
SE	− 4.251	1.051	− 4.330	1.183	− 4.232	1.451	0.391
Cylinder	0.741	0.632	0.712	0.668	0.701	0.421	0.401
Coma	0.100	0.056	0.095	0.042	0.089	0.051	0.233
Trefoil	0.100	0.070	0.089	0.077	0.093	0.091	0.071
SA	0.069	0.065	0.063	0.065	0.070	0.068	0.561
RMS-HOA	0.287	0.065	0.278	0.064	0.251	0.078	0.488
After intervention							
SE	− 4.312	1.300	− 4.210	1.436	− 4.221	1.641	0.105
Cylinder	0.801	0.532	0.769	0.768	0.771	0.700	0.100
Coma	0.167	0.086	0.135	0.082	0.119	0.101	0.032
Trefoil	0.123	0.091	0.100	0.097	0.113	0.075	0.055
SA	0.084	0.075	0.093	0.055	0.090	0.088	0.288
RMS-HOA	0.327	0.085	0.299	0.084	0.344	0.058	0.045

Dioptric spherical equivalent values standardized for a 5 mm pupil were applied throughout

95% repeatability limit, *SE* Spherical equivalent, *SA* Spherical aberration, *RMS-HOA* Root mean square higher order aberrations

## Discussion

Previous studies have shown that HOA are caused not only by corneal irregularities but also by tear film disorders [5]. Regular administration of lubricating eye drops is generally recommended prior to refractive surgery, especially if dry eye is suspected [4]. To date, there is contradictory evidence as toward the influence of different AT supplements on HOA and it seems to depend on their main lubricant component. An improvement of HOA has been reported after application of 0.25% polyethylene glycol and / or 0.1% hyaluronic acid [15, 16], while HOA increased in dry and healthy eyes after application of 0.3% hypromellose [17]. In this context, viscous eye drops induce more short-term HOA [18]. While several factors such as pupil diameter can influence HOA measurements [19], the volume of eye drops instilled also plays an important role in the HOA development and visual performance [20]. To avoid this bias, we instructed participants to administer only a single eye drop. Our study is one of the first studies to investigate the relationship between the semifluorinated alkane (SFA), F6H8 eye drops, and HOA. These water- and preservative-free eye drops facilitate the supply of oxygen to the cornea, which may also improve dry eye symptoms [21]. We showed that HOA increase following the use of F6H8 four times daily for four weeks. However, in our previous work, we did not demonstrate any significant effects of F6H8 on HoA in the short- and long-term period using the Pentacam Scheimpflug tomograph [22]. The difference in the devices used could explain this variation in the results.

Repeatability of HOA measurements is critical to assess its effects on visual quality. Cheng et al. [23] demonstrated fluctuations in HOA over time, which they attributed in part to tear film instability, and therefore recommended against relying solely on averaged RMS values. However, no clinically significant differences were found over time in HOA measurements in untreated eyes in different study groups, indicating high repeatability [24, 25]. Other works showed high precision and excellent repeatability of the WASCA aberrometer when assessing HOA [26, 27]. We demonstrated higher repeatability of HOA measurements after application of F6H8 than in untreated eyes, which is in accordance with the results of previously mentioned works. Several studies have proven the efficacy of F6H8 in the treatment of dry eye symptoms [28–30]. In a preclinical trial, Agarwal et al. [31] showed a beneficial effect of F6H8 by immediately fusing with the precorneal tear film and compensating for surface irregularities. Schmidl et al. [32] confirmed these results using a modified AS-OCT system. They showed a stabilization of the tear film expressed in tear film thickness (TFT). This regularization of the corneal surface and stabilization of the tear film could explain the increase in measurement accuracy demonstrated in our current study. On the one hand, we noticed an increase of HOA after long-term application of F6H8, which could be related to the lower tear evaporation with F6H8 [33]. On the other hand, its regulating effects on the corneal surface leads to increased repeatability of the obtained refractive and aberrometric values. The negative effect of HOA increase on visual quality after use of AT has been shown previously [34]. In other studies, 3 and 1.3% of patients treated with F6H8, respectively, complained of blurred vision (0.3% in the control groups) [35, 36]. However, the participants in our study did not report any disturbances, possibly due to the minimal changes in HOA, which were measured but not clinically perceived. The correlation between the F6H8-associated increase of HOA and (subjective) visual quality is an interesting topic to investigate in the future, as different AT may show different effects.

Variations in HOA are multifactorially influenced. Blinking and pre-existing refractive errors can dynamically influence HOA assessment [37, 38]. After blinking, aberrations occur after an average of 2.9 s as a sign of premature tear break up, while these are found in healthy individuals only after 6.1 s [39]. Ridder et al. [40] have explained contradictory effects of lubricants on aberrations in several studies with differences in the point of time of the measurement after the blink.

For the management of dry eye symptoms and in terms of measurement accuracy when using the WASCA aberrometer for refractive assessment before and after refractive surgery, F6H8 seems to be a suitable option. Especially for wavefront-guided ablations, accurate aberrometric data are essential for optimal surgical results. However, comparison of refractive values with other refraction methods could clarify these conclusions, but this is beyond the purpose of this study. Regarding the treatment of postoperative dry eye symptoms, F6H8 may not be beneficial because of HOA enhancement.

The following summarizes the limitations of our study: As mentioned above, the integrity and stability of the tear film depend on many factors, including exogenous ones. Hence, the quality of the tear film may also fluctuate during measurement of aberrations. Therefore, it is practically impossible to differentiate between these factors. In addition, different devices for measuring HOA can only be compared with each other to a limited extent. In a study comparing several aberrometers, the Visual Function Analyzer (Tracey Technologies, Houston Texas, USA) deviated significantly from other aberrometers in the measurement of RMS values [41]. Furthermore, our investigations were performed only on healthy eyes of young individuals. However, it would be interesting to evaluate in future work the effect of F6H8 on HOA in dry eye patients. Additionally, transfer of these results to elderly patients (e.g., cataract patients) should be done with caution. Finally, we did not directly measure the increase of the lipid layer thickness (LLT). This may be a limitation, but since F6H8 was administered based on a fixed study protocol, a causality between a possible increase in lipid layer and an increase in HOA analyzed seems very likely. Besides, measurements of the lipid layer have many limitations, because F6H8 and water have a similar refractive index and both mix to from a transparent fluid, leading to difficulties in distinguishing the different layers of the tear film [32, 42].

In conclusion, the results of our study show that administration of single drop F6H8 induces HOA when applied continuously for four weeks. HOA are likely to be triggered by lipid-based tear supplements. Additionally, the reliability of measurement of HOA and refractive data is more accurate when lipid-based tear supplements are used. We believe that these results are important for refractive surgeons and that future studies with larger cohorts and more assessment factors would be a valuable addition to this work.

## References

[1] Messmer EM (2015). The pathophysiology, diagnosis, and treatment of dry eye disease. Dtsch Arztebl Int.

[2] Sambhi RS, Sambhi GDS, Mather R, Malvankar-Mehta MS (2020). Dry eye after refractive surgery: a meta-analysis. Can J Ophthalmol.

[3] Raoof D, Pineda R (2014). Dry eye after laser in-situ keratomileusis. Semin Ophthalmol.

[4] D'Souza S, James E, Swarup R (2020). Algorithmic approach to diagnosis and management of post-refractive surgery dry eye disease. Indian J Ophthalmol.

[5] Koh S (2016). Mechanisms of visual disturbance in dry eye. Cornea.

[6] Montes-Mico R, Cervino A, Ferrer-Blasco T (2010). The tear film and the optical quality of the eye. Ocul Surf.

[7] Rhee J, Chan TC-Y, Chow SS-W (2022). A systematic review on the association between tear film metrics and higher order aberrations in dry eye disease and treatment. Ophthalmol Therapy.

[8] Koh S (2018). Irregular astigmatism and higher-order aberrations in eyes with dry eye disease. Invest Ophthalmol Vis Sci.

[9] Montés-Micó R, Cáliz A, Alió JL (2004). Wavefront analysis of higher order aberrations in dry eye patients. J Refract Surg.

[10] Lee SY, Tong L (2012). Lipid-containing lubricants for dry eye: a systematic review. Optom Vis Sci.

[11] Hagyo K, Csakany B, Lang Z, Nemeth J (2009). Variability of higher order wavefront aberrations after blinks. J Refract Surg.

[12] Buhren J, Kohnen T (2007). Application of wavefront analysis in clinical and scientific settings. From irregular astigmatism to aberrations of a higher order–part I: basic principles. Ophthalmologe.

[13] Craig JP, Nichols KK, Akpek EK (2017). TFOS DEWS ii definition and classification report. Ocul Surf.

[14] Jones L, Downie LE, Korb D (2017). TFOS DEWS II management and therapy report. Ocul Surf.

[15] Lu N, Lin F, Huang Z (2016). Changes of corneal wavefront aberrations in dry eye patients after treatment with artificial lubricant drops. J Ophthalmol.

[16] Montes-Mico R, Cervino A, Ferrer-Blasco T (2010). Optical quality after instillation of eyedrops in dry-eye syndrome. J Cataract Refract Surg.

[17] McGinnigle S, Eperjesi F, Naroo SA (2014). A preliminary investigation into the effects of ocular lubricants on higher order aberrations in normal and dry eye subjects. Cont Lens Anterior Eye.

[18] Berger JS, Head KR, Salmon TO (2009). Comparison of two artificial tear formulations using aberrometry. Clin Exp Optom.

[19] Carkeet A, Velaedan S, Tan YK (2003). Higher order ocular aberrations after cycloplegic and non-cycloplegic pupil dilation. J Refract Surg.

[20] Kaido M, Ishida R, Dogru M (2008). Efficacy of punctum plug treatment in short break-up time dry eye. Optom Vis Sci.

[21] Stolowich N, Vittitow J, Kissling R, Borchman D (2023). Oxygen-carrying capacity of perfluorohexyloctane, a novel eye drop for dry eye disease. Curr Ther Res.

[22] Habbe KJ, Frings A, Saad A, Geerling G (2023). The influence of a mineral oil cationic nanoemulsion or perfluorohexyloctane on the tear film lipid layer and higher order aberrations. PLoS One.

[23] Cheng X, Himebaugh NL, Kollbaum PS (2004). Test-retest reliability of clinical Shack-Hartmann measurements. Invest Ophthalmol Vis Sci.

[24] Miranda MA, O'Donnell C, Radhakrishnan H (2009). Repeatability of corneal and ocular aberration measurements and changes in aberrations over one week. Clin Exp Optom.

[25] López-Miguel A, Martínez-Almeida L, González-García MJ (2013). Precision of higher-order aberration measurements with a new Placido-disk topographer and Hartmann-Shack wavefront sensor. J Cataract Refract Surg.

[26] Cervino A, Hosking SL, Dunne MC (2007). Operator-induced errors in Hartmann-Shack wavefront sensing: model eye study. J Cataract Refract Surg.

[27] Cerviño A, Hosking SL, Montés-Micó R (2008). Comparison of higher order aberrations measured by NIDEK OPD-Scan dynamic skiascopy and Zeiss WASCA Hartmann-Shack aberrometers. J Refract Surg.

[28] Steven P, Scherer D, Krösser S (2015). Semifluorinated alkane eye drops for treatment of dry eye disease–a prospective, multicenter noninterventional study. J Ocul Pharmacol Ther.

[29] Steven P, Augustin AJ, Geerling G (2017). Semifluorinated alkane eye drops for treatment of dry eye disease due to meibomian gland disease. J Ocul Pharmacol Ther.

[30] Delicado-Miralles M, Velasco E, Díaz-Tahoces A (2021). Deciphering the action of perfluorohexyloctane eye drops to reduce ocular discomfort and pain. Front Med (Lausanne).

[31] Agarwal P, Khun D, Krosser S (2019). Preclinical studies evaluating the effect of semifluorinated alkanes on ocular surface and tear fluid dynamics. Ocul Surf.

[32] Schmidl D, Bata AM, Szegedi S (2020). Influence of perfluorohexyloctane eye drops on tear film thickness in patients with mild to moderate dry eye disease: a randomized controlled clinical trial. J Ocul Pharmacol Ther.

[33] Vittitow J, Kissling R, DeCory H, Borchman D (2023). In vitro inhibition of evaporation with perfluorohexyloctane, an eye drop for dry eye disease. Curr Ther Res Clin Exp.

[34] Koh S, Maeda N, Ikeda C (2013). Effect of instillation of eyedrops for dry eye on optical quality. Invest Ophthalmol Vis Sci.

[35] Sheppard JD, Kurata F, Epitropoulos AT (2023). NOV03 for signs and symptoms of dry eye disease associated with meibomian gland dysfunction: the randomized phase 3 MOJAVE study. Am J Ophthalmol.

[36] Tauber J, Berdy GJ, Wirta DL (2023). NOV03 for dry eye disease associated with meibomian gland dysfunction: results of the randomized phase 3 GOBI study. Ophthalmology.

[37] Chen X, Lu Y, Dai JH, Wang L (2009). Agreement measurement of ocular wavefront aberrations with three different aberrometers. Zhonghua Yan Ke Za Zhi.

[38] Koh S, Maeda N, Hirohara Y (2006). Serial measurements of higher-order aberrations after blinking in normal subjects. Invest Ophthalmol Vis Sci.

[39] Montes-Mico R, Alio JL, Charman WN (2005). Dynamic changes in the tear film in dry eyes. Invest Ophthalmol Vis Sci.

[40] Ridder WH, LaMotte J, Hall JQ (2009). Contrast sensitivity and tear layer aberrometry in dry eye patients. Optom Vis Sci.

[41] Rozema JJ, Van Dyck DE, Tassignon MJ (2006). Clinical comparison of 6 aberrometers. Part 2: statistical comparison in a test group. J Cataract Refract Surg.

[42] Liang Y, Kociok N, Leszczuk M (2008). A cleaning solution for silicone intraocular lenses: "sticky silicone oil". Br J Ophthalmol.

